# Influence of internal migration on reproductive health in Myanmar: results from a recent cross-sectional survey

**DOI:** 10.1186/s12889-016-2915-2

**Published:** 2016-03-09

**Authors:** May Sudhinaraset, Nadia Diamond-Smith, May Me Thet, Tin Aung

**Affiliations:** Global Health Group, Global Health Sciences, University of California, San Francisco, 550 16th Street, San Francisco, CA 94116 USA; Department of Epidemiology and Biostatistics, University of California, San Francisco, USA; Population Services International, Myanmar, No. 16, Shwe Gon Taing, Street 4, Yangon, Myanmar

**Keywords:** Migration, Reproductive health, Myanmar, Family planning, Urban health, Maternal health, Antenatal care, Delivery, Internal migration

## Abstract

**Background:**

Maternal and reproductive health remains a significant public health issue in Myanmar. Little data exists on women’s health issues, including social and demographic influences. While past studies have demonstrated rural/urban health disparities, an increasingly important population resulting from urban growth in Myanmar is the internal migrant population, individuals moving within the country for better job or educational opportunities. Past studies suggest that women make up more than half of internal migrants, yet there is a dearth of information on this new wave of migration, particularly on women’s reproductive health issues. The objective of this study is to assess the influence of women’s migration in Myanmar on reproductive health outcomes, including delivering in a facility, using a skilled birth attendant, and using a modern method of family planning.

**Methods:**

Data from a cross-sectional household survey using multistage cluster sampling design conducted between September to October 2014 was used to assess the accessibility and the use of maternal and child health products and services. A total of 1800 currently married women of reproductive age, including 348 from urban and 1452 from rural areas, were recruited to complete surveys. A set of multivariable regressions was performed to assess reproductive health outcomes and predictors.

**Results:**

Across health indicators, female migrants had better health outcomes compared to non-migrants. Controlling for demographic characteristics, migrants were 1.60 times more likely to use a modern form of family planning compared to non-migrants (*p* < 0.01) and use antenatal care during pregnancy (*p* < 0.05). While not statistically significant, migrants were 1.29 times more likely to deliver with a skilled attendant and 1.08 times more likely to deliver in a facility.

**Conclusions:**

This study found that female migrants in Myanmar reported better health outcomes compared to non-migrant women in regards to family planning and maternal health. Future research should focus on monitoring the outcomes of migrants and their children over time to assess long-term impacts.

## Background

Urbanization and internal migration have recently been gaining attention in Myanmar due to economic and political changes. In 2011, Myanmar witnessed a significant moment in its history in which the country transitioned into a democratic government, opening up for the first time to international markets. The country experienced an increase in foreign investment and aid, and urban growth [1]. While past studies have demonstrated rural/urban health disparities, an increasingly important population resulting from urban growth is the internal migrant population, individuals moving within the country for better job or educational opportunities. Much of the migration literature in Myanmar to date has highlighted stressful migratory flows, including a significant literature on refugee settlements on the Thai/Myanmar border [2, 3], environmental migration due to natural disasters [4], and ethnic conflict and tension [5]. However, these migratory flows may no longer reflect the new wave of migration occurring today in Myanmar. Studies suggest that women are making up an increasingly large proportion of migrants, with more than half of internal migrants (54 %) being women [6]; however, there is a dearth of information on this new wave of migration, particularly on women’s reproductive health issues.

To date, one of the richest sources of data for migration in Myanmar is the Fertility and Reproductive Health Surveys (FRHS), conducted between 1991 and 2007. Data indicates that 14 out of 100 people moved in 2007 compared to only 10 out of 100 in 1991. Like other parts of the world, young people ages 20–24 years represent the majority of recent migrants. Women in particular are more likely than men to move to join their families or work in the agricultural sector, with marriage migration being the most common reason for migrating [6]. There are limitations in the data, however. First, this data is from 2007 and given recent political and social movements, updated migration data is needed. Second, few studies have attempted to examine differences across migration status in Myanmar related to women’s health, including family planning, delivery, and antenatal care. Data on internal migration, particularly among women, is limited in Myanmar.

The literature on how migration influences sexual and reproductive health is mixed. Studies from other Asian contexts find that internal migrants are less likely to use antenatal care [7], and experience higher rates of maternal mortality and unintended pregnancy [8–10] compared to their non-migrant counterparts. Internal migrants face a number of challenges including lower education status, discrimination, social isolation, conflicts between traditional and modern city values, and increased sexual opportunities [11]. However, data also supports a so-called “healthy migrant effect,” which shows that migrants generally exhibit better health outcomes compared to their non-migrant counterparts, and that migrants are selected for health; that is, individuals must be healthier in general to be able to migrate [12]. Over time, there is a convergence among receiving communities and migrant health outcomes. In addition to health selection effects, strong social networks in destination communities may also improve health service utilization. In Italy, for example, strong informational and social support was associated with increased health utilization [13]. Given the increase of urban growth and mobile populations, it is important to examine the patterns of reproductive health outcomes across migration status to inform urban programs and policies.

Maternal and reproductive health issues remain a significant public health issue in Myanmar. The burden of infant mortality rates is one of the highest in the region, with reports as high as 105 per 1000 live births [14]. While cities may have more service points than rural areas, often times reproductive health services remain inaccessible to migrants and slum-dwellers, particularly those who are young and unmarried [15]. Long travel and wait times, overburdened facilities, and costs all remain barriers to care. Other challenges faced by the urban poor in Myanmar include misinformation about sexual and reproductive health issues, widespread use of traditional forms of contraception, harmful traditional practices during deliveries, and common practices of unsafe abortions [15]. The objective of this study is to assess the influence of women’s migration in Myanmar on reproductive health outcomes, including delivering in a facility, using a skilled birth attendant, and using a modern method of family planning.

## Methods

### Data collection and procedures

**Fig. 1:**
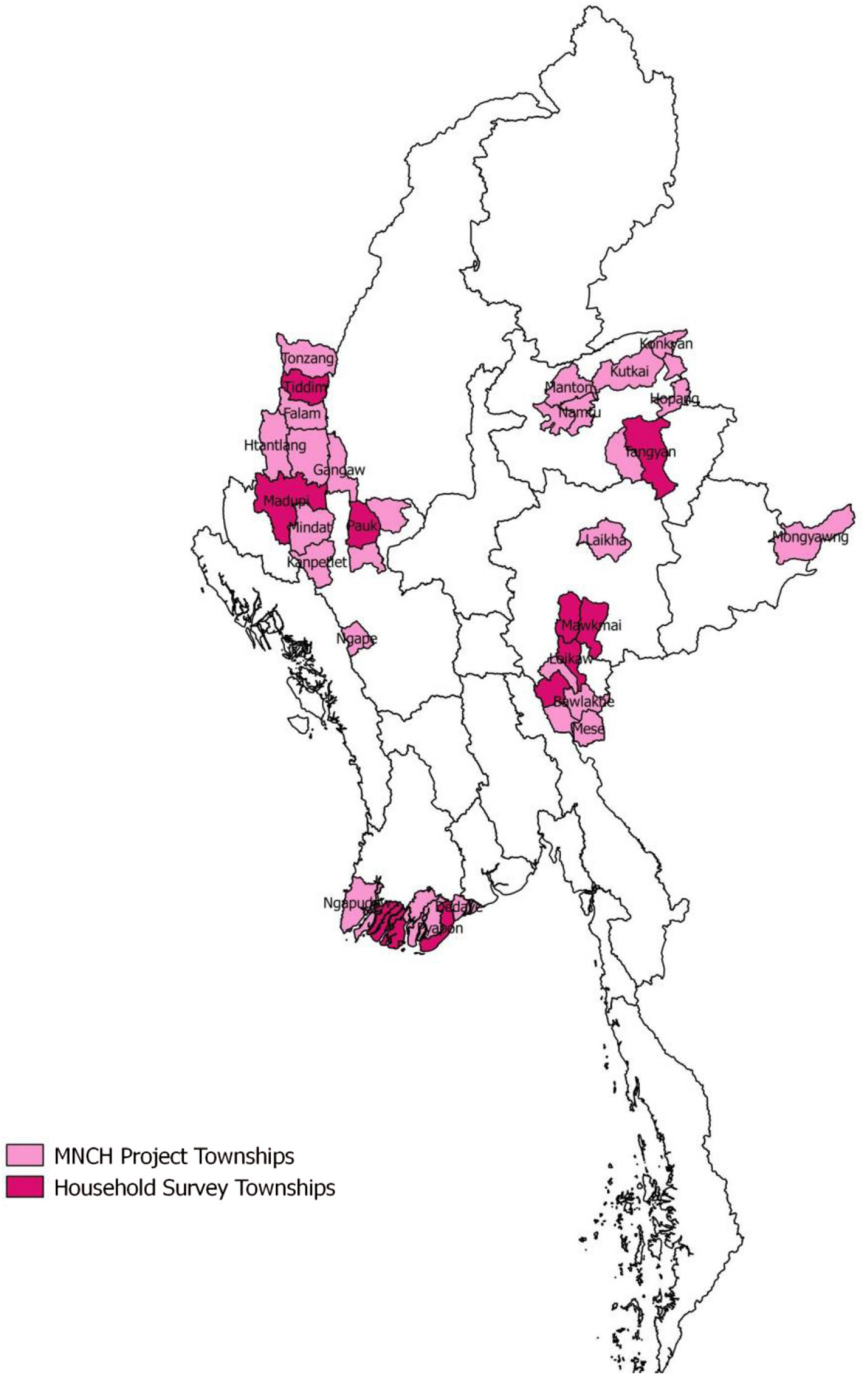
Map of study sites

We used data from a cross-sectional household survey using multistage cluster sampling design conducted between September to October 2014 to assess the accessibility and the use of maternal and child health products and services. In the first stage, ten townships out of 42 project townships were selected using a Probability Proportional to Size (PPS) method (see Fig. 1). A township consisted of urban and rural areas. The population in a township is approximately split into 20 % urban and 80 % rural. In the second stage, within a township, two urban wards and four village tracts were selected. In the third stage, after mapping, 17 households from each urban ward and 36 household from each village tract were selected by systemic random method. A total of 1800 currently married women of reproductive age (348 from urban and 1452 from rural areas) were recruited through face-to-face interviews.

### Measures

A modified form of the Multiple Indicator Cluster Survey (MICS) questionnaire in Myanmar 2009–2010 was used for the study. The questionnaire was translated into Myanmar and back translated into English. The main predictors of interest included urban/rural status, which was categorized using data from the Myanmar Information Management Unit (MIMU). The second main predictor, migration status, was coded as non-migrants, who were those who reported that they had always lived in their current area and migrants, who reported that they had not always lived in their current area. The first main outcome was current family planning use, which was based on a question asking “Are you currently doing something or using any method to delay or avoid getting pregnant?” and thus included both modern and traditional family planning methods. The second outcome of interest was whether the women reported seeing anyone for antenatal care during their last pregnancy. The third outcome of interest was whether a woman delivered with a skilled attendant at her last delivery. Skilled attendants included doctors, nurses or midwives, compared to unskilled, which included traditional birth attendants, community health workers, family members, or no one. The final outcome of interest was the place of delivery being home or a facility. Facilities included anything other than the respondent’s home or someone else’s home, including government hospitals, clinics and health centers, and private hospitals and clinics.

A series of individual and household variables were also included in the models. These included the woman’s age groups (18–24 years, 25–30 years, 31–39 years and 40–49 years), education (illiterate/none, primary, secondary and university), age at marriage (<18 years, 18–24 years, 25–30 years, 31–39 years and 40–49 years) and her parity (continuous). A wealth quintile score was constructed using principal components analysis, and included questions about the household’s type of toilet, source of water, source of fuel, and construction material of the walls of the house [16].

A number of analyses were conducted to assess differences between migrants and non-migrants. First, using chi-2 statistics, we assessed differences between migrants and non-migrants across basic demographic characteristics. Second, we employed multivariable logistic regression to control for potential confounders. For the first model exploring family planning use (the dependent variable), only non-pregnant women were included. This model tests whether there are differences across migrant and non-migrant women (the main predictor variable) in regards to family planning use. Family planning use was a binary variable (yes/no). For the last three models for delivery and antenatal care (each modeled separately as binary variables), only women who had had a birth in the last 2 years were included. These models also test whether there were differences among migrants vs. non-migrants in relation to delivery and antenatal care outcomes. Data was analyzed using STATA/SE version 12 [17].

### Ethical approval

The study and all study materials were reviewed and approved by the Institutional Review Board at Population Services International in Myanmar. All women were at least 18 years of age. Researchers first approached women, explained the objectives and study procedures, and participants were asked whether they would be interested in participating in the study. Verbal informed consent was obtained from all study participants. The data is housed at Population Services International in Myanmar.

## Results

### Demographic characteristics

In total, there were 1800 women who responded to the survey, including 1556 non-migrants and 244 migrants (see Table 1). Approximately 13.0 % of women were between 18 and 24 years old, 23.2 % were 25–30 years, 32.3 % were 31–39 years, and 31.6 % were 49–49 years. There were differences across migrant and non-migrant status, but the majority of both were older than 30 years of age. There were no statistically significant differences across education status or age of marriage, with approximately 29 % having no formal education, approximately 28.7 % with primary education, 37.9 % with secondary education, and only 4.0 % with university education. The overwhelming majority of women married before the age of 25 years of age (83.3 %). Migrants were significantly more likely to be of higher socioeconomic status compared to non-migrants (39.3 % vs. 17.0 %, *p* = 0.000). Approximately 81.0 % of the sample lived in an urban area. Migrants were more likely to live in urban areas compared to non-migrants (34.8 % vs. 16.9 %, *p* = 0.000).

**Table 1:** Demographics by migrant/non-migrant status

	Non-migrant (%) (*n* = 1556)	Migrant (%) (*n* = 244)	Total (%) (*N* = 1800)	*p*-value
Age of respondent				
18–24 years	13.1	11.9	12.9	0.005
25–30 years	23.4	21.7	23.2	
31–39	30.8	41.8	32.3	
40–49	32.7	24.6	31.6	
Education status				
Illiterate/no formal education	29.9	26.2	29.4	0.287
Primary	29.0	26.6	28.7	
Secondary	37.3	41.8	37.9	
University	3.8	5.3	4.0	
Age of marriage				
<18 years	19.9	23.4	20.4	0.686
18–24 years	63.2	61.1	62.9	
25–30 years	14.3	13.1	14.2	
31–39	2.2	2.5	2.3	
40–49	0.3	0.00	0.2	
Number of living children				
0–1	24.1	20.6	23.7	0.010
2–3	44.7	53.6	45.9	
4–5	20.2	20.6	20.3	
6+	11.0	5.2	10.2	
Wealth quintiles				
Lowest quintile	21.9	9.8	20.2	0.000
Lower quintile	20.6	15.2	19.8	
Middle quintile	20.5	16.4	19.9	
Higher quintile	20.1	19.3	20	
Highest quintile	17.0	39.3	20	
Are you currently doing something or using any method to delay or avoid getting pregnant?				
No	65.0	49.6	62.9	0.000
Yes	35.0	50.4	37.1	
Attendant at delivery				
TBA, CHW, no one, family	52.5	32.9	49.7	0.002
Doctor, nurse, midwife	47.5	67.1	50.3	
Place of delivery				
Home	81.1	72.9	80	0.109
Facility	18.9	27.1	20	
Did you see anyone for antenatal care for that pregnancy?				
No	29.6	8.6	26.6	0.000
Yes	70.4	91.4	73.4	
Residency				
Rural	83.1	65.2	80.7	0.000
Urban	16.9	34.8	19.3	

Abbreviations: *TBA* (traditional birth attendant), *CHW* (community health worker)

In regards to health outcomes, compared to non-migrants, migrants were more likely to indicate that they were currently using a method of contraception to delay or avoid getting pregnant (50.4 % vs. 35.0 %, *p* = 0.000), deliver with a doctor, nurse or midwife (67.1 % vs. 47.5 %, *p* = 0.002), deliver in a facility, (although this was not statistically significant with 27.1 % vs. 18.9 %, *p* = 0.109), and receive antenatal care (91.4 % vs. 70.4 %, *p* = 0.000).

Of the individuals who migrated, the majority of migration occurred in the context of marriage. Over 55 % of individuals moved because of marriage, approximately 24 % moved because of employment for themselves or family members, another 17.6 % moved with family, and almost 3 % moved for other reasons, such as education opportunities or living in a city. Overwhelmingly, female migrants were younger –approximately 70 % were younger than 24 years of age, with 22 % migrating between 18 years, and 47 % migrating between 18 and 24 years of age. Approximately 55 % migrated from rural to rural areas and 25 % from rural to urban cities (see Table 2).

**Table 2:** Reasons for migration among those that migrated (*N* = 244)

Reason for migration	(*N*=244) (%)
Marriage	135 (55.3)
Employment for myself or family	59 (24.2)
Moved with family	43 (17.6)
Other: education, city	7 (2.9)
Age at Migration	
<18	53 (21.7)
18–24 years	114 (46.7)
25–30 years	43 (17.6)
31–39 years	32 (13.1)
40–49 years	2 (0.8)
Type of Migrant	
Rural-rural	135 (55.3)
Rural–urban	60 (24.6)
Urban-urban	25 (10.3)
Urban–rural	24 (9.8)

### Multivariable results

To assess whether there were urban/rural disparities, the study used multivariate regression to explore predictors of family planning use, antenatal care, using a skilled attendant, and delivering in a facility (see Table 3). The study found that compared to rural populations, individuals living in urban areas had lower odds (odds ratio (OR) = 0.86) of using a current family planning methods, but increased odds of using antenatal care (OR = 2.73, *p* < 0.1), delivering with a skilled attendant (OR = 3.48, *p* < 0.01), and delivering in a facility (OR = 1.51, *p* > 0.1), although only skilled attendance was statistically significant.

**Table 3:** Multivariate logistic regression of urban/rural status on indicators of family planning and maternal health care utilization

	Current family planning use OR (SE)	Any antenatal care OR (SE)	Skilled attendant OR (SE)	Deliver at facility OR (SE)
Urban vs. rural	0.86	2.73*	3.48***	1.51
	(0.13)	(−1.44)	(−1.2)	(−0.47)
Years of education	1.07	1.90***	1.38***	1.49**
	(0.07)	(−0.27)	(−0.17)	(−0.24)
Age in years	0.93	1.06	1.71***	1.32
	(0.06)	(−0.2)	(−0.28)	(−0.27)
Age at marriage	0.81**	0.66**	0.78	1.02
	(0.07)	(−0.13)	(−0.14)	(−0.22)
Wealth quintile	1.56***	2.02***	1.57***	1.61***
	(0.07)	(−0.23)	(−0.14)	(−0.18)
Parity	0.80***	0.84**	0.68***	0.67***
	(0.03)	(−0.07)	(−0.06)	(−0.08)
Constant	0.44***	0.57	0.31***	0.05***
	(0.09)	(−0.2)	(−0.11)	(−0.03)
Observations	1602	489	489	489

***p < 0.01, **p < 0.05, *p < 0.1

Other variables in the model suggest that women with higher education were more likely to use any antenatal care, skilled attendant, and deliver in a facility (*p* < 0.01). Age was only associated with using a skilled attendant, with older women having increased odds compared to younger women (OR = 1.71). Older age of marriage was associated with both lower use of family planning and using antenatal care. Women with a greater number of living children had lower odds of currently using family planning (OR = 0.80), *p* < 0.01). Wealth quintile, not surprisingly, was highly associated across all four family planning and maternal health outcomes (*p* < 0.01), while higher parity was associated with lower odds of use of any of the four health outcomes.

Across health indicators, female migrants had better health outcomes compared to non-migrants (see Table 4). Controlling for demographic characteristics, migrants had increased odds of using a modern form of family planning compared to non-migrants (OR = 1.60, *p* < 0.01) and antenatal care during pregnancy (OR = 2.61, *p* < 0.05). While not statistically significant, migrants were more likely to deliver with a skilled attendant (OR = 1.29) and more likely to deliver in a facility (OR = 1.08).

**Table 4:** Multivariate logistic regression of migrant and urban/rural status on indicators of family planning and maternal health care utilization

	Current family planning use OR (SE)	Any antenatal care OR (SE)	Skilled attendant OR (SE)	Deliver at facility OR (SE)
Migrant vs. non-migrant	1.60***	2.61**	1.29	1.08
	(0.26)	(1.26)	(0.41)	(0.36)
Urban vs. rural	0.82	2.33	3.38***	1.51
	(0.13)	(1.23)	(1.17)	(0.47)
Years of education	1.08	1.94***	1.39***	1.49**
	(0.07)	(0.28)	(0.17)	(0.24)
Age in years	0.93	1.03	1.69***	1.31
	(0.06)	(0.20)	(0.28)	(0.27)
Age at marriage	0.82**	0.68**	0.79	1.03
	(0.07)	(0.13)	(0.14)	(0.23)
Wealth quintile	1.54***	1.95***	1.55***	1.60***
	(0.07)	(0.22)	(0.14)	(0.18)
Parity	0.80***	0.85**	0.68***	0.67***
	(0.03)	(0.07)	(0.06)	(0.08)
Constant	0.43***	0.54*	0.31***	0.05***
	(0.08)	(0.19)	(0.11)	(0.03)
Observations	1602	489	489	489

***p < 0.01, **p < 0.05, *p < 0.1

In regards to other demographic characteristics, individuals in urban areas were more likely to deliver with a skilled attendant (OR = 3.38, *p* < 0.01). As expected, as individuals are more educated, they are also more likely to receive any antenatal care, deliver with a skilled attendant, and deliver in a facility. Age of the respondent was only associated with delivering with a skilled attendant (OR = 1.69, *p* < 0.01). Surprisingly, higher age of marriage was associated with lower use of family planning and antenatal care. Women of higher socioeconomic status were more likely to use family planning, antenatal care during pregnancy, skilled attendant at birth, and deliver in a facility. Women with more children are less likely to use family planning, antenatal care, skilled attendant, or to deliver in a facility.

## Discussion

This study found that female migrants in Myanmar report better health outcomes compared to non-migrant women in regards to family planning and maternal health. Myanmar is witnessing rapid political and economic reform, and while the migration literature has overwhelmingly focused on negative aspects of migration, this study suggests that there are protective influences to migration and mobility. Migrants were more likely to use a modern form of contraception and use antenatal care compared to non-migrants. There are a number of potential explanations for the improved health outcomes. For example, improved health strong informational support network in the destination community may improve knowledge on where to seek care [13] compared to non-migrants who may not have these established ties. Social support in their sending communities [18–20] may contribute to improved psychosocial outcomes and thus health utilization patterns. Other migratory streams suggests a Healthy Migrant Effect [21–23], in which migrants report better health outcomes when they first arrive to their destination; however, over time and across generation status, migrant health deteriorates as they continue to live in their new society. It is thus important for future research to monitor the outcomes of migrants and their children over time to see if this occurs in Myanmar as well.

Results also suggest that there is an urban advantage to maternal health services, specifically for use of antenatal care and delivery with a skilled attendant. This is unsurprising given that urban centers have a higher number of delivery points and therefore services are more easily accessible for maternal health care [24]. It is interesting to note that after controlling for migration status, urban residence only confers a benefit for delivery with a skilled attendant, and not for antenatal care use. This might suggest that urban areas are especially more likely to provide skilled providers for all women, not only migrants who might be more motivated or differ in other ways from the rest of the urban population. One surprising result was that women in urban areas were slightly less likely to use family planning, but this was only marginally significant. This is corroborated by other studies that find that while access points for reproductive health services may exist, that vulnerable populations, including younger and unmarried women, may not be able to reach these delivery points due to transportation, cost, and social barriers [15].

Moreover, this study also highlights differences between migrants and non-migrants in regards to demographic characteristics. Migrant women were more likely to be aged 31–39 years, which is contrary to other studies that find that migrant women are typically younger (i.e. 20–24 years). One possible explanation for this is that we ask about lifetime migration rather than recent migration (most commonly defined as less than 5 years living in their new place of residence). Migrants were also more likely to be in the highest wealth quintile compared to non-migrants, suggesting that migrants are being selected for higher levels of socioeconomic status. Like other studies, the data also suggests that marriage migration was the most common reason for migration [25]. Most people migrated in their late adolescence through the early years of adulthood. Additionally, most of the migrants in this sample migrated between rural areas or from rural to urban areas, as would be expected given the sampling nature of the study and patterns of increased urbanization in a mostly rural country.

This study has several limitations. First, this is a cross-sectional study, thus we are only able to explore associations. However, in most cases it is likely that the migration event occurred before delivery or use of family planning, since such a large proportion of women migrated for marriage and there is little childbearing outside of marriage in this setting. Additionally, we are limited in that we only have data on currently married women, and not on their husbands or other family members that might be important decision makers about place or attendant at delivery or family planning use. For example, the behaviors of rural women who migrate for marriage and marry urban men who have never migrated might look very different from similar women who migrate with their rural husbands to urban areas. Since there were relatively few migrants compared to non-migrants in the study, it was not possible to tease out different types of migrants. For example, individuals migrating from rural areas compared to other urban areas have been shown to be less likely to use contraception [26]. However, this study assessed differences between rural and urban health outcomes, and while urban women reported slightly better health outcomes, it was not consistent across all health outcomes. Therefore, this suggests that migration, and not just place of birth, may be an influential factor in it of itself. Results of the study are generally reflective of other townships across the country, including reasons for migration. In Myanmar, historically women tend to migrate because of marriage; however, more recently, women also migrate for better life opportunities such as job and education. Duration of being a migrant has also been shown to influence health outcomes, as individuals begin to acculturate to their destination communities [27]. We assessed duration effects and found no statistically significant results. We left these findings out of this manuscript because the null results may have been due to the low numbers of migrants in the sample.

Despite these limitations, these findings are important because they use migration data linked to women’s health outcome in Myanmar. Data from Myanmar are particularly limited, and it is a particularly pivotal point in the country’s history to begin to document women’s migration experiences. Future studies should oversample migrants to better understand differences between migrant types as well as the influence of age of migration. Globally, urbanization has increased dramatically, and of increasing importance in Myanmar. Rural agricultural society is giving way to urban cities, and Myanmar is on the precipice of this phenomenon. Therefore, this research is extremely timely, especially given the lack of data from the country on migration and women’s health and the large number of female migrants. Information on where women deliver and use of family planning is important for policy-makers and health administrators, as they plan for providing services to changing demographics and increasing populations.

## Conclusion

Reproductive health remains a public health issue in Myanmar, reporting one of the highest rates of maternal mortality and neonatal mortality in the region. Family planning use remains low, and services are needed for vulnerable populations, including unmarried, adolescent, and mobile populations. This study found that female migrants in Myanmar are generally healthier in regards to family planning use and delivery compared to non-migrants. These results are timely given urban growth in the country and lack of data collected on mobile populations, particularly women moving from rural to urban areas due to labor, educational, or marriage opportunities. Future research efforts should continue to monitor this population closely, given other countries that have documented the decline in health over time for migrants and their children.
